# The Role of Viruses in the Pathogenesis of Immune-Mediated Gastro-Intestinal Diseases

**DOI:** 10.3390/ijms25158301

**Published:** 2024-07-30

**Authors:** Francesca Bernardi, Federica Ungaro, Ferdinando D’Amico, Alessandra Zilli, Tommaso Lorenzo Parigi, Luca Massimino, Mariangela Allocca, Silvio Danese, Federica Furfaro

**Affiliations:** 1 Gastroenterology and Endoscopy, IRCCS Ospedale San Raffaele, 20132 Milan, Italy; bernardi.francesca@hsr.it (F.B.); ungaro.federica@hsr.it (F.U.); damico.ferdinando@hsr.it (F.D.); zilli.alessandra@hsr.it (A.Z.); parigi.tommaso@hsr.it (T.L.P.); massimino.luca@hsr.it (L.M.); allocca.mariangela@hsr.it (M.A.); danese.silvio@hsr.it (S.D.); 2 Gastroenterology and Endoscopy, Vita-Salute San Raffaele University, Via Olgettina, 58, 20132 Milan, Italy

**Keywords:** virus, virome, celiac disease, inflammatory bowel disease, Crohn’s disease, ulcerative colitis, achalasia

## Abstract

Immune-mediated gastrointestinal (GI) diseases, including achalasia, celiac disease, and inflammatory bowel diseases, pose significant challenges in diagnosis and management due to their complex etiology and diverse clinical manifestations. While genetic predispositions and environmental factors have been extensively studied in the context of these conditions, the role of viral infections and virome dysbiosis remains a subject of growing interest. This review aims to elucidate the involvement of viral infections in the pathogenesis of immune-mediated GI diseases, focusing on achalasia and celiac disease, as well as the virome dysbiosis in IBD. Recent evidence suggests that viral pathogens, ranging from common respiratory viruses to enteroviruses and herpesviruses, may trigger or exacerbate achalasia and celiac disease by disrupting immune homeostasis in the GI tract. Furthermore, alterations in the microbiota and, specifically, in the virome composition and viral–host interactions have been implicated in perpetuating chronic intestinal inflammation in IBD. By synthesizing current knowledge on viral contributions to immune-mediated GI diseases, this review aims to provide insights into the complex interplay between viral infections, host genetics, and virome dysbiosis, shedding light on novel therapeutic strategies aimed at mitigating the burden of these debilitating conditions on patients’ health and quality of life.

## 1. Introduction

Immune-mediated gastrointestinal (GI) diseases encompass a spectrum of disorders defined by imbalanced immune responses targeting the GI tract, resulting in inflammation and tissue damage [1,2,3]. Immune-mediated damage can affect one or more organs of the gastrointestinal system: the esophagus is involved in achalasia [4] and eosinophilic esophagitis [5], the stomach is involved in autoimmune gastritis [6], the duodenum and small intestine are affected in celiac disease [7], immunoglobulin-associated enteropathy [8] and the colon are involved primarily in ulcerative colitis [1], the bile ducts are compromised in primary sclerosing cholangitis and primary biliary cholangitis [9,10], and the liver is affected in autoimmune hepatitis [11]. Additionally, Crohn’s disease [1], Behçet’s vasculitis [12], and eosinophilic gastroenteritis [13] can damage the entire gastrointestinal tract.

Among these conditions, celiac disease and achalasia stand out as prime examples, each presenting unique challenges in diagnosis and management [2,3]. While considerable research has focused on genetic predispositions and environmental triggers for these diseases, emerging evidence suggests a potential role for viral infections in their pathogenesis [14,15].

Celiac disease, an autoimmune condition activated by the ingestion of gluten in genetically predisposed subjects, presents with a wide array of gastrointestinal and extra-intestinal manifestations [7]. Similarly, achalasia, a rare esophageal motility disorder defined by impaired lower esophageal sphincter relaxation and the absence of peristalsis, poses significant clinical complexities [4]. Despite advancements in understanding their etiology, the precise mechanisms underlying achalasia and celiac disease remain elusive, necessitating exploration into novel contributing factors. Recent studies have implicated viral infections as potential triggers for the onset or exacerbation of celiac disease and achalasia: viral pathogens, ranging from common respiratory viruses to *herpesviruses* and *enteroviruses*, have been proposed to disrupt normal immune homeostasis in the GI tract, thereby precipitating immune-mediated damage [14,16,17,18,19]. Moreover, the interplay between viral infections and host genetic susceptibility may further potentiate disease development in susceptible individuals [4,16,18,20,21].

In addition to viral infections, alterations in the virome, the viral component of the intestinal microbiota, have garnered attention in the pathogenesis of immune-mediated GI diseases, particularly inflammatory bowel diseases (IBDs) [22]. Dysbiosis of the virome, characterized by shifts in viral populations and perturbations in viral–host interactions, has been linked to the perpetuation of chronic intestinal inflammation in IBDs [22,23]. Understanding the intricate balance between viral diversity and host immune responses is crucial for elucidating the role of the virome in IBD pathogenesis and exploring possible therapeutic interventions.

In light of these considerations, this review aims to provide a comprehensive overview of the current understanding of viral contributions to the pathogenesis of immune-mediated GI diseases, with a specific focus on celiac disease, achalasia, and IBDs. By elucidating the complex interplay between viral infections, host genetics, and dysbiosis of the virome, we endeavor to shed light on novel avenues for the prevention and management of these conditions.

### Intestine Immune Response to Viral Infection

**Figure 1:**
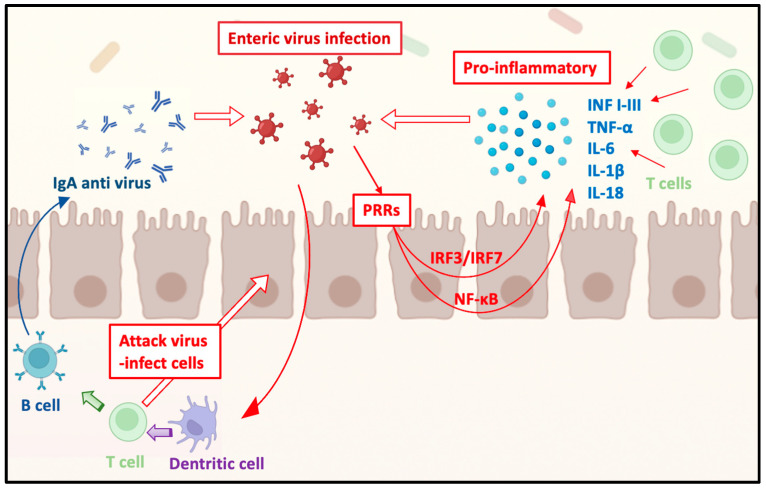
Intestine immune response to viral infection.

The importance of the immunological function of the gastrointestinal tract is now well recognized. The intestinal mucosa, consisting of an epithelial cell layer, underlying lamina propria, and muscular layer, houses numerous immune cells primarily located within its two most superficial layers [24]. Moreover, the presence of Peyer’s patches and associated mesenteric lymph nodes facilitates the immune system’s organized responses to antigens encountered by the intestinal mucosa [24]. Intestinal epithelial cells and innate immune cells can activate immune responses against potentially pathogenic antigens via pathogen recognition receptors (PRRs) on their surfaces or within the cytosol [25]. In cases of enteric viral infections, the PRRs involved typically include RNA or DNA sensors such as TLR3, TLR-9, RLRs, cGAS, and STING 25 [25]. Upon activation, these receptors initiate signaling cascades through the NF-κB or IRF3/IRF7 pathways, resulting in the production of pro-inflammatory cytokines such as Type I-III interferons, TNF-α, IL-6, IL-1β, and IL-18, which recruit leukocytes [26]. Simultaneously, effector and memory T lymphocytes are activated in the lymph nodes following the presentation of viral antigens by dendritic cells in the lamina propria via MHC molecules [27]. These differentiated T cells can attack virus-infected cells and subsequently activate B cells in Peyer’s patches, leading to the differentiation of plasma cells that produce anti-virus IgA, exerting their function within the intestinal lumen [27] (Figure 1). Maintaining a balance in this environment between pathogen protection and immune tolerance is critical to prevent excessive inflammation and consequent immune-mediated diseases.

## 2. Celiac Disease

Celiac disease (CD) is an immune-mediated gastrointestinal disease that exhibits a global distribution, with its highest incidence observed in regions predominantly inhabited by individuals of European ancestry, where prevalence rates range from 1% to 2%.

CD presents as a multifaceted gastrointestinal disorder with autoimmune characteristics, typically manifesting in genetically susceptible subjects who express human leukocyte antigen (HLA) class II molecules, particularly HLA-DQ8 or HLA-DQ2 [20]. Notable diagnostic indicators include the presence of antibodies targeting tissue transglutaminase 2 (TG2) and deamidated gluten peptides, which serve as robust markers of disease activity [28].

The pathological hallmark of CD involves the inflammation and architectural distortion of the intestinal microvilli, triggered by an aberrant immune response directed against gluten proteins (gliadin) [29]. This breakdown of immune tolerance to gluten is prominently characterized by the activation of CD4 T cells that specifically respond to gluten peptides in association with HLA-DQ2 or HLA-DQ8 molecules [29]. Concurrently, there is a pronounced expansion of cytotoxic CD8+ intraepithelial lymphocytes within the intestinal mucosa that contributes to the destruction of intestinal epithelial cells [29]. These mechanistic insights underscore the prevailing notion that CD mainly represents an immune disorder mediated by T cells, wherein dysregulated immune responses orchestrated by gluten-reactive CD4 and CD8 lymphocytes precipitate tissue damage and clinical symptomatology [29].

The determinants influencing the onset of clinical manifestations in disease presentation are intricate, often involving a convergence of environmental factors [20]. These factors encompass various elements such as the timing and dosage of initial gluten exposure, occurrences of common childhood viral infections, and alterations in the microbiome composition [14,30,31].

The significance of environmental influences in disease pathogenesis has been so much acknowledged that they have been reported to act synergistically along with genetic predisposition, which is an element fundamental to the disease onset but not sufficient to promote the pathogenesis per se [7], as we discuss in the following sections.

### The Role of Viruses in Celiac Disease

In genetically predisposed individuals, contact with the prolamin fraction of gluten, gliadins, induces immune responses locally within the gastrointestinal tract and systemically [32]. Moreover, aside from genetic factors, additional environmental elements, such as prior exposure to prevalent infections like *reovirus*, *enterovirus*, and, potentially, other microbial agents may also have a role in disease pathogenesis [14,33]. Studies indicate that common viral agents can incite inflammatory reactions to gluten antigens by favoring TH1 immune responses over Treg responses [14]. This shift in immune balance has been linked to the development of symptomatic CD in genetically predisposed individuals [14]. Another significant factor concerns the frequent production of type 1 interferons (IFN-1) during viral infections [34], which disrupt oral tolerance and promote the development of celiac disease (CD) in murine models [35,36]. In humans, the administration of IFNα has been linked to the onset of CD [35]. Additionally, type 1 IFNs play essential roles within the network of genes associated with susceptibility to CD [20]. Moreover, infections caused by *adenovirus*, *enterovirus*, *hepatitis C virus*, and *rotavirus* have been correlated with an elevated incidence of CD [17,33,37].

*Reoviridae* infections are frequently encountered and typically exhibit nonpathogenic behavior; however, certain members of this viral family, notably *rotaviruses*, have been associated with severe manifestations such as diarrhea and abdominal distress in pediatric populations [16]. Studies have identified elevated occurrences of *rotavirus* gastroenteritis among children diagnosed with CD [17], prompting investigations into the potential preventive effects of vaccines for *rotavirus* against the development of CD [38].

Findings from a prospective study involving nearly 2000 children genetically susceptible to CD suggested that frequent occurrences of *rotavirus* infection may heighten the risk of developing CD [17]. *Rotavirus*, via a process of molecular mimicry, has been postulated to potentially instigate CD and type 1 diabetes mellitus among children with genetic predisposition, although the precise role of molecular mimicry as a trigger for CD remains inconclusive and lacks robust evidence [39]. Nevertheless, vaccinations targeting *rotavirus* have shown favorable safety profiles and may even confer protection against the development of CD [40,41]. *Rotavirus* infections have been implicated in directly augmenting mucosal permeability within the gastrointestinal tract, underscoring another correlation between *rotavirus* infections and CD [42]. Notably, the viral protein VP7, an integral component of the *rotavirus* outer layer, has been scrutinized and gene array analyses have revealed that purified anti-rotavirus VP7 antibodies possess the capacity to modulate genes associated with apoptosis and inflammation, while also exerting influence over the integrity of the epithelial barrier within intestinal epithelial cells, phenomena commonly observed in CD pathology [43].

*Reoviruses* have been implicated in promoting enterocyte apoptosis and potentially initiating a pro-inflammatory response to ingested food antigens [44]. Pivotal research utilizing recombinant human viruses and transgenic rodents with genetic susceptibility to CD highlighted the ability of the *reovirus* TL1 strain (Lang type) to induce CD pathology: TL1 was shown to hinder the differentiation of peripherally induced regulatory T cells and stimulate TH1 immunity against dietary antigens at the initiation sites of oral antigen responses [14]. The investigation identified the upregulation of IRF1 (interferon regulatory factor 1), a factor also elevated in the mucosa of children with CD [14]. Additionally, mesenteric lymph node dendritic cells from TL1-infected mice failed to induce TH1 cell differentiation in the absence of IRF1 expression [14]. Notably, in mice expressing HLA-DQ8, TL1 infection led to the presence of tissue transglutaminase 2 (TGA2) [14].

Another study explored the association between *reoviruses* and gut physiology, suggesting that the T1L strain could manipulate apoptosis pathways to activate the inflammatory cascade, disrupt immune tolerance to dietary proteins, and induce pro-inflammatory phenotypes in dendritic cells [44]. The M2 gene, encoding an outer-capsid protein, was identified as potentially influential in intestinal pathogenesis [44]. Furthermore, it was elucidated that T1L can evade antiviral responses, establishing permanent infection and inducing the release of type 1 interferons, stimulating IRF-1 expression in the lamina propria [44]. This inflammatory milieu facilitates the dendritic cell-mediated presentation of new food antigens in the mesenteric lymph nodes, ultimately activating gluten-specific TH1 cells [44]. The increased expression of type 1 interferons in T1L infection suppresses regulatory T cells, fostering TH1 immunity to gluten and contributing to the development of CD [44].

An additional study examined 100 samples, half from CD patients, to detect anti-*reovirus* antibodies, revealing a link between reoviral infections and CD, along with an increase in the type 1 interferon pathway [45]. The investigation suggested that type 1 interferons may have elucidated the loss of oral tolerance in a group of IL15-negative patients and proposed that viral infections could incite a pro-inflammatory reaction in the mucosa of the small intestine, culminating in the breakdown of tolerance towards oral antigens [45]. Moreover, NK cells could be implicated in the tolerance breakdown, as their depletion hinders the T1L-induced loss of tolerance to newly introduced food antigens in mice [46].

*Noroviruses*, as single-stranded RNA viruses, propagate within host organisms through the fecal–oral transmission pathway, constituting a significant etiological agent of gastroenteritis [47]. Murine *norovirus* CW3 VP1 prompts a TH1 immune response to dietary antigens: the loss of dietary antigen tolerance is orchestrated by dendritic cells, particularly the CD103+, CD11b+, and CD8α+ subsets, which predominantly capture dietary ovalbumin (OVA) [48]. The study demonstrated a shared transcriptional signature induced by *norovirus* and *reovirus* T1L in mesenteric lymph nodes, indicative of tolerance breakdown: IRF-1 was upregulated at the site of dietary antigen response determination [14,48]. Notably, mice lacking IRF1 exhibited skewed dendritic cells but not activation and IL12 production in CD103+, CD11b+, and CD8α+ dendritic cells [48].

Research has illuminated a potential association between *enterovirus* infection in children, verified through viral seroconversion or stool analysis, and CD, confirmed by duodenal biopsies [33,49,50]. During *enterovirus* infection, particularly with *echovirus 16*, transglutaminase (TGA) may be induced: the inflammation in the small intestine could lead to heightened tissue TGA levels at inflamed sites [51]. Another possible nexus between *enterovirus* and CD involves zonulin, a controller of tight junctions [52]. Elevated zonulin levels have been observed in CD patients, correlating with the concentration of *enterovirus* in the small intestinal mucosa exhibiting severe atrophic changes [52].

Of note, *enterovirus* may also be associated with *parechovirus* in triggering CD in genetically susceptible children [53]. An investigation entailed the detection of viral PCR in monthly fecal specimens collected from children with retrospective TGA testing performed on earlier blood samples [53]. The findings revealed a higher incidence of *parechovirus* preceding the development of TG2 antibodies, particularly when concomitant with *enterovirus* [53].

Studies conducted on the contribution of *Epstein–Barr virus* (*EBV*) to the genesis of CD are limited and involve only a small number of patients [51]. However, this virus may play a role in exacerbating refractory CD: by examining PCR in duodenal biopsies of individuals with refractory CD, *EBV* was identified in 70% of cases, compared to only 16% of patients with typical CD [54]. The mechanism by which the virus exacerbates the disease may lie in its continuous reactivation of pro-inflammatory cells, which hinder the healing of the intestinal epithelium [54].

Conversely, *Citomegalovirus* (*CMV*) appears to play a safeguarding role against the disease: in a study involving 1068 patients from the Generation R cohort (a prospective, population-based cohort study involving patients from the gestational period to young adulthood), it was demonstrated that patients with subclinical CD exhibited a reduction in T Vδ1+ cells and, simultaneously, a rise in CD57+ cells [55]. Conversely, in subjects with positive *CMV* serology, Vδ1+ cells increased; thus, CMV may be protective against CD [55]. Nevertheless, since the molecules activating Vδ1+ and CD57+ cells are not identified, it is not possible to establish a direct link (Table 1).

It is well established within the scientific community that the *Hepatitis C Virus* (*HCV*) is correlated with autoimmune diseases [56]. Multiple lines of evidence indicate that TGA levels can elevate during *HCV* infection [51]. Additionally, CD has been observed to be at least twice as prevalent among patients with HCV-related cirrhosis [57]. A clinical investigation demonstrated that adhering to a gluten-free diet leads to improvements in liver function tests among *HCV* patients [57]. Conversely, another study highlighted that patients exhibiting TGA prior to commencing *HCV* treatment with interferons were at a heightened risk of developing CD during the treatment regimen [58]. Explorations into the molecular interplay between CD and *HCV* through transcriptional regulatory networks unveiled a specific amalgamation of genes spanning various functional categories implicated in inflammation, such as NFKB1, STAT3, IRF1, interleukins, and chemokines [37]. A comparative analysis involving 321 CD-associated genes and 1032 HCV-associated genes was conducted to elucidate shared pathways [37]. Utilizing computational techniques, 11 transcription factors were pinpointed as pivotal molecules in this context [37]. While the precise mechanistic link between *HCV* and CD remains incompletely understood, emerging research indicates that HCV might play a role in the onset of CD in individuals exhibiting positive serological markers for the virus.

A few studies have found a correlation between CD and infections caused by *influenza virus* [59] and *respiratory syncytial virus* [60]. However, the pathogenetic mechanism by which these microorganisms would lead to the onset of the disease has not yet been elucidated. Further research is needed to delve deeper into the topic.

## 3. Achalasia

Achalasia represents a primary esophageal motility disorder defined by the gradual degeneration of myenteric neurons, resulting in the impairment of peristalsis and inadequate relaxation of the lower esophageal sphincter [61]. Clinically, it manifests through symptoms such as dysphagia for liquids and solids, regurgitation, chest pain, and weight loss [61]. The condition exhibits a prevalence of eight cases per million individuals and an estimated annual incidence ranging from 0.3 to 1.63 per 100,000 individuals [62]. It is further classified into three subtypes established by distinctive manifestations observed in high-resolution manometry: type I, classic achalasia; type II, exhibiting panesophageal pressurization; and type III, spastic achalasia [63].

The pathophysiology of achalasia entails the depletion of inhibitory enteric neurons, leading to the release of nitric oxide and vasoactive intestinal polypeptides within the esophageal myenteric plexus [4]. Initially identified as a degenerative disorder of the myenteric plexus, subsequent investigations revealed inflammatory cell infiltration within the myenteric ganglia in individuals with achalasia [64]. Further studies have implicated immunological factors, viral infections, and host genetics in the pathogenesis of achalasia [15]. Immunological mechanisms emerge as pivotal, with viral infections potentially triggering dysregulated immune responses in susceptible individuals harboring specific immunogenetic variations (for example, HLA-DRB1*14:54, DQB1*05:03, and the conserved haplotype DRB1*14:54-DQB1*05:03 in Mexicans confer risk for the development of achalasia) [65].

### The Role of Viruses in Achalasia

While emerging evidence suggests an association between the etiology of achalasia and inflammatory-mediated neuronal loss, the precise trigger for T cell-mediated infiltration and subsequent attacks on these neurons remains unknown [66]. Viral infection has been hypothesized as a primary causative factor based on preliminary indications [66].

The *herpesvirus* class displays a preference for squamous epithelium and comprises neurotropic viruses [67]. *Herpes simplex virus* (*HSV*) and *varicella-zoster virus* (*VZV*) can establish latent infections within neurons [67]. This characteristic is speculated to be linked to injury within the esophageal myenteric plexus in achalasia [18].

A significant study revealed that only T cells from achalasia patients exhibit activation in response to *HSV-1* antigens [18]. Incubation with *HSV-1* antigens resulted in T cell proliferation and the production of Th1-type cytokines, particularly interferon-gamma (IFN-γ) [18]. Additionally, the study noted a limited expression of T-cell receptor β variable gene families (2–5 out of 26) and a Gaussian-like distribution of β-chain complementarity determining region 3 (CDR3) length spectra types of lymphocytes in 41 achalasia patients and 23 controls [18]. This observation suggests clonal expansion through the oligoclonal selection of T cells in achalasia [18]. When coupled with functional data, this indicates that a subgroup of T cells remains continuously activated, most likely by *HSV-1* or HSV-1-like antigen moieties [18]. This process could be implicated in most patients, given that anti-HSV-1 antibodies and HSV-1 DNA were found in 84% and 63% of cases, respectively [18].

In support of this, further studies demonstrate how stimulation through *HSV-1* in the LES tissue and blood immune cells of patients with achalasia leads to an increase in the production of IFN-γ, cytokines, and lymphocytes, resulting in an inflammatory reaction [64,68]. Additionally, a study showed the presence of HSV-1 DNA and RNA in the LES tissues of patients with achalasia, in contrast to controls, where polymerase chain reaction (PCR), reverse transcription–polymerase chain reaction (RT-PCR), and immunohistochemistry did not detect viral DNA or RNA [64].

The presence of VZV DNA has also been discerned in tissue specimens obtained from individuals afflicted with achalasia, in stark contrast to control subjects [19]. Furthermore, the aforementioned study illuminated that the concentration of anti-VZV antibodies was markedly elevated in achalasia patients compared to their counterparts in the control group [19]. Naik et al. elucidated that individuals with achalasia manifest heightened incidence rates of *VZV* infection: salivary viral DNA was detected in 80% of the patients, with transcripts encoding VZV late gene products observed in 87% of tissues subjected to myotomy [21]. They posited that the virus, harbored latently within esophageal neurons, underwent reactivation, thereby instigating a chronic infection that elicited perturbations in the functional regulation of esophageal motility, concomitantly impinging upon the control mechanisms governing lower esophageal sphincter contractility [21].

*HIV* is a neurotropic virus capable of leading to autonomic innervation loss, which may account for cases of achalasia in HIV-positive patients [69] and the amelioration of symptoms following antiretroviral and antitubercular therapy [70]. Concurrently, the condition of AIDS may facilitate opportunistic viruses such as *CMV* and *HSV-1* to induce the onset of achalasia-specific symptoms [71]. Further insights are required to better comprehend this subject matter (Table 2).

## 4. Inflammatory Bowel Diseases

Inflammatory bowel diseases (IBD) constitute a group of chronic inflammatory disorders regarding the gastrointestinal tract, comprising Crohn’s disease and ulcerative colitis (UC) [72]. These conditions are characterized by periods of active inflammation interspersed with remission phases [73].

The global incidence and prevalence of IBDs have shown an upward trend in recent decades, particularly in Westernized countries, with emerging pockets of incidence in traditionally low-prevalence regions [74]. Notably, the peak age of onset occurs during early adulthood, although cases can manifest at any age [74].

Clinical manifestations of IBDs encompass a spectrum of gastrointestinal and extraintestinal symptoms [73]. Common gastrointestinal symptoms include abdominal pain, diarrhea, rectal bleeding, and weight loss, often accompanied by systemic features such as fatigue and malaise [73]. Extraintestinal manifestations could compromise joints, skin, eyes, and other organ systems, further contributing to the heterogeneous clinical presentation of IBDs [75].

IBDs are immune-mediated gastrointestinal diseases, believed to stem from environmental exposures impacting a genetically susceptible host [76]. These environmental factors facilitate immune dysregulation in genetically predisposed individuals, eliciting an abnormal response that culminates in enduring bowel inflammation and, in certain instances, inflammation beyond the intestinal tract [76]. Various environmental risk factors, encompassing drug exposure (antibiotics), viral infections, psychological stress, air pollutants, dietary patterns, and chemical agents, have been associated with IBD pathogenesis [77]. However, despite some evidence, current data remain inconclusive in establishing definitive causal relationships: all these factors could contribute to the development of IBDs, each in its own way and with its own significance, given its multifactorial genesis.

### 4.1. The Role of Viruses in IBDs

Unlike the pathogenesis described in the addressed conditions, the literature emphasizes the role of viruses within the intestinal virome in the development of IBDs, rather than as isolated infections that trigger the diseases [78]. Viruses, together with bacteria, archaea, protists, and fungi, are part of the intestinal microbiota, which outnumber prokaryotes in the gut by tenfold [79]. Virus significance lies in their capacity to interact with both bacteria (bacteriophages) and host cells (eukaryotic-targeting viruses), thereby affecting the overall intestinal homeostasis [79].

Intestinal dysbiosis, characterized by an altered composition of commensal gut microorganisms, has been associated with the pathogenesis of IBDs [78]. Changes in the composition of the gut virome have been linked to the initiation and severity of IBDs: metagenomic analyses have revealed an increased abundance of tailed bacteriophages (*Caudovirales*) and a reduction in spherical *Microviridae* accompanied by an overall decline in bacterial and virome diversity [22,23], observed in both pediatric ileal Crohn’s disease [80] and very early onset IBDs [81]. Moreover, eukaryotic-targeting viruses, owing to their capacity to interact with both immune and non-immune cells of the host, have been implicated in the initial stages of intestinal inflammation, indicating a possible role in IBD pathogenesis. Indeed, elevated levels of the eukaryotic *Orthohepadnavirius* genus-encoded transcripts were more abundant in treatment-naive patients with UC compared to those with Crohn’s disease and healthy controls, and, additionally, individuals with Crohn’s disease exhibited an increased prevalence of *Hepeviridae* and a decreased prevalence of *Virgaviridae* families [82]. Of note, another study identified a positive correlation between the prevalence of the eukaryotic *Anelloviridae* family in early-onset IBD patients undergoing immunosuppressive treatment [81].

**Figure 2:**
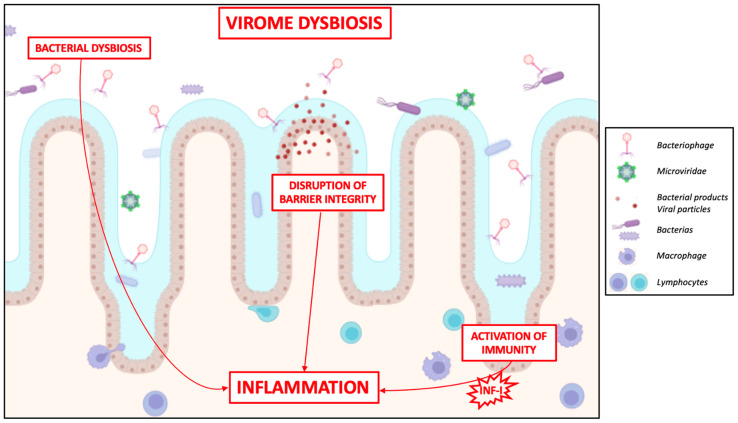
Alterations in the gut virome are associated with the initiation and severity of IBDs. Virome changes impact intestinal mucosa integrity: phages indirectly stimulate the immune response by releasing bacterial products following lysis or transcytosis, which activate pattern recognition receptors on IECs or immune cells. Moreover, virome factors disrupt barrier integrity and influence intestinal physiology through interactions with commensals. Phages modify host bacterial dynamics, leading to dysbiosis and contributing to IBD pathogenesis. Furthermore, phages shape immunity, prompting type-I interferon responses in immune cells, thereby exacerbating intestinal inflammation and disease severity.

The virome dysbiosis may contribute to IBD pathogenesis through various pathways, including the regulation of gut bacterial populations, alterations in intestinal barrier integrity, and, consequently, the modulation of pro-inflammatory actions and local immune responses (Figure 2).

#### 4.1.1. Regulation of Gut Bacterial Populations

Phages have the capacity to influence the population dynamics of host bacteria, thereby precipitating changes in the abundance of specific bacterial species, determining a whole microbiota dysbiosis, underlying IBD pathogenesis [83]. Indeed, the high abundance of *Caudovirales* bacteriophages in IBDs, concomitant with reduced bacterial richness and diversity, contributes to dysbiosis of the overall microbiota and its attendant consequences [84]. A study elucidated an augmented abundance of phages infecting *Bacteroides uniformis* and *Bacteroides thetaiotaomicron*, coupled with a diminished abundance of their host bacteria in IBD patients, underscoring a discernible relationship between phage abundance and host bacterial populations [85]. Furthermore, a metagenomic investigation revealed a heightened prevalence or abundance of phages infecting *Faecalibacterium prausnitzii*, a beneficial commensal, in the stools of IBD patients compared to healthy controls [86]. Given the documented decrease in *Faecalibacterium prausnitzii* in IBD patients, it is posited that phages may incite or exacerbate the depletion of *Faecalibacterium prausnitzii* through enhanced phage-mediated mortality [86]. Comprehensive experimental inquiries are warranted to elucidate the intricate interactions between phages and bacteria in the context of IBDs.

#### 4.1.2. Alterations in Intestinal Barrier Integrity

The modulation of the virome can exert influence on the integrity of the intestinal mucosa, a stratified tissue consisting of diverse cell types that collectively coordinate pathways, engage in synergistic signaling, and communicate with one another and with the commensal microbiota adhering to it to maintain homeostasis [87]. The intestinal epithelial layer comprises tight junction proteins linking a monolayer of columnar intestinal epithelial cells alongside specialized cells such as goblet and Paneth cells, pivotal components of the innate immune response [88]. Acting as the primary barrier, it serves to restrict excessive interaction between immune cells and microorganisms, thereby shielding the gut from unwarranted immune reactions [87]. Upon the arrival or penetration of intestinal commensals to the epithelial surface, immune system activation occurs, instigating the characteristic cascade of events underlying IBD pathogenesis [87].

The literature demonstrates that alterations in the virome can indeed affect the integrity of the intestinal mucosa [89]. Phages have the capacity to indirectly stimulate the immune response by releasing bacterial products subsequent to bacterial lysis or translocation across the epithelium (transcytosis), thereby activating pattern recognition receptors on intestinal epithelial cells or resident immune cells [89]. Additionally, specific factors derived from the virome can disrupt barrier integrity and influence intestinal physiology, often through engagement with other commensals [89]. For instance, Sinha et al. conducted a study in which they pooled viral-like particles (VLPs) from UC patients, predominantly enriched in *Microviridae* phages and partially in crAss-like, *Siphoviridae*, and *Podoviridae phages*, and transplanted them into human microbiota-associated mice [90]. The transplantation of UC VLPs aggravated the severity of colitis [90].

Particularly noteworthy is the viral protein Hepatitis B X (HBx), encoded by the *Orthohepadnavirus* genus, which disarranges the intestinal epithelial barrier by inducing the dedifferentiation of epithelial cell tasks, ultimately leading to alterations in the immune microenvironment and intestinal inflammation in vivo [91]. Evidence indicates that the colonization of the gut virome by the Orthohepadnavirus genus is linked to the pathogenesis of UC in both pediatric and adult populations [91].

Certain viruses appear to modulate goblet cell function: *Enterovirus 71* (*EV71*) infects goblet cells, reducing the expression of goblet cell-derived mucins MUC1 and MUC2, indicating a viral-induced alteration of goblet cell function [92]. Moreover, human adenovirus has been demonstrated to especially infect goblet cells, with this favoritism being strain-specific: adenovirus species C exhibits goblet cell tropism [93]. Additionally, astrovirus VA1 (AstV-VA1) infects goblet cells, while murine astrovirus (MuAstV) targets actively secreting goblet cells and may gain advantages from enhanced mucus production in reaction to goblet cell infection, potentially leading to goblet cell exhaustion and a weakened mucus barrier, culminating in inflammation or secondary bacterial infection [94]. Such events can contribute to UC, which has been correlated with a dysfunctional colonic mucus layer and a decreased number of mucin-producing goblet cells, facilitating increased contact between the microbiota and the epithelium, thereby promoting further inflammation [95].

#### 4.1.3. Modulation of Immune Responses

It is widely recognized that IBD is characterized by a dysregulation of immune response and inflammatory processes.

Phages have been proposed as a significant factor in shaping innate, humoral, and cell-mediated immunity [96]. Regarding innate immunity, a study has demonstrated that filamentous Pf bacteriophages produced by *Pseudomonas aeruginosa* are internalized by dendritic cells, macrophages, and B-cells, leading to the induction of type-I interferon responses, thus enabling infection by the associated bacteria [97].

On the other side, in vitro experiments have suggested that dendritic cell recognition of phage DNA can stimulate IFN-γ production through a Toll-like receptor 9-dependent pathway, thereby exacerbating intestinal inflammation and contributing to disease severity [98].

Notably, current research indicates that viral infections with enteric viruses enhance the proliferation of specific immune cell populations in the intestine, including colonic and small intestinal lamina propria leukocytes, comprising effector memory T cells, macrophages, and plasmacytoid DCs [99].

Furthermore, similar to bacteria, phages can traverse the intestinal barrier and migrate to lymphoid tissue, peripheral blood, and internal organs, where they can directly influence the host immune system [100].

Ultimately, current data regarding the impact of gut phages on IBDs through immune modulation are limited. The concept proposed above is grounded in the immune dysregulation observed in IBDs, the alteration of the gut virome observed in the diseases, and the demonstrated ability of phages to regulate immune responses, but further investigations are warranted.

## 5. Clinical Implications of Host–Virus Interaction: Challenges and Future Perspectives

Despite evidence demonstrating that viral infections may contribute to the development of immune-mediated gastrointestinal diseases, particularly serving as triggers for celiac disease and achalasia, the relationships explored between various infections and the onset of pathology are not yet straightforward and a definite cause–effect relationship is lacking [38,64]. Consequently, there is still no literature-based evidence indicating whether vaccination against these viruses may or may not play a protective role in the development of these diseases. It would be of interest to conduct studies to investigate whether certain vaccinations against specific viruses could be protective in the development of celiac disease or achalasia.

Regarding IBD pathogenesis, recent persuasive data have underscored the pivotal roles of intestinal microbiota in precipitating immune dysregulation and inflammation, thus prompting the investigation of microbial-targeted therapies for IBD treatment [101]. Probiotics, prebiotics, and fecal microbiota transplantation (FMT) are widely employed for such therapies, having demonstrated both safety and potential efficacy in ameliorating dysregulated immune responses [102,103]. Additionally, phage therapy, representing another efficacious approach for reinstating gut microbiome equilibrium, has emerged as a therapeutic modality for IBD treatment [104]. Phages have historically served as a treatment modality for bacterial infectious diseases, leveraging their ability to lyse host bacteria [105]. Consequently, phages have been employed in IBDs to address specific bacterial colonization or infections, including adherent-invasive *E. coli*, Klebsiella pneumoniae, Clostridioides difficile, and colorectal cancer associated with Fusobacterium nucleatum [106,107].

Particularly, adherent-invasive *E. coli* (AIEC) strains have been notably prevalent in Crohn’s disease patients compared to healthy individuals, with studies implicating AIEC strains in perpetuating intestinal inflammation in IBDs [106,108,109]. Several investigations have demonstrated the efficacy of phages in decreasing intestinal *E. coli* colonization: through the isolation of selected phages targeting enteropathogenic *E. coli* found in hospital sewage, researchers showed that a unique dose of phage cocktail (2 × 10^9^ PFU/mL) effectively controlled bacterial infection [110]. Additionally, current research employing phage therapy against the AIEC strain in a dextran sulfate sodium (DSS)-induced colitis mouse model evidences a significant reduction in AIEC strain LF82 colonization and the alleviation of symptoms over a 2-week period following oral treatment with a phage cocktail for a single day [111]. Furthermore, in a double-blinded, placebo-controlled crossover trial, the administration of a commercially available cocktail of bacteriophages targeting Escherichia coli for 28 days demonstrated a selective reduction in fecal Escherichia coli loads without inducing global disruption to the gut microbiota community [112]. Concurrently, this intervention led to an elevation in anti-inflammatory cytokines, specifically interleukin-4 (IL-4) [112].

Despite promising results from preclinical animal model studies, several safety and regulatory issues must be addressed before widespread clinical applications of phage therapies. Although early-phase clinical trials have demonstrated the tolerability of oral phage administration in humans, these studies were conducted in individuals with a healthy gut milieu [113,114,115]. Amidst inflammation, the bacteriophage-mediated lysis of pathogens could result in the release of pathogen-associated molecular patterns within the gastrointestinal tract, potentially exacerbating inflammatory reactions [87,116]. Thus, the safety profile of phage therapy warrants careful consideration, and the timing of administration poses a challenge for future studies.

Given the documented modification of the gut virome in IBD patients, researchers have begun investigating novel therapeutic strategies able to modify the virome, such as fecal virome transplantation (FVT), which entails the transfer of solely gut viruses from healthy donors into afflicted patients [117]. Clinical trials of FVT in IBDs remain limited, though preliminary investigations have shown promise: fresh sterile fecal filtrates containing the fecal virome alleviated symptoms of patients with Clostridium difficile infection and resulted in sustained symptom remission for at least 6 months post-transplantation [118]. Subsequently, studies in animal models have demonstrated the potential of FVT to ameliorate DSS-induced colitis, as evidenced by the reduction of inflammatory markers in the plasma and the amelioration of symptoms in mice [119].

Further corroborating the substantial impact of virome alteration on inflammation exacerbation, a recent study demonstrated that transplanting the virome of patients with UC into mice intensified the severity of DSS colitis [90]. This exacerbation manifested in shortened colon length and the elevated production of pro-inflammatory cytokines (TNF-α and IL-1β) [90]. Notably, the study ruled out the influence of endotoxin in the virome preparations, such as lipopolysaccharide, as evidenced by reduced colitis severity in mice administered heat-killed virome compared to those receiving intact virome from UC patients [90].

Despite the potential therapeutic role that FVT may demonstrate, considering these data, the long-term efficacy and effects on the bacteriome remain uncertain, warranting further investigation to elucidate the specific taxa and mechanisms underlying the therapeutic potential of the virome in IBDs. Consequently, progress in developing virome-based therapies hinges on a comprehensive understanding of how viruses influence host metabolism and contribute to both diseased and healthy gut states.

## 6. Conclusions

The intricate interplay between viral infections and immune-mediated gastrointestinal diseases, such as CD, achalasia, and IBDs, underscores the need for further research and therapeutic innovation in this field. While the precise mechanisms underlying viral contributions to achalasia and celiac disease pathogenesis remain to be fully elucidated, the absence of established preventive measures emphasizes the urgency of continued investigation into viral triggers and host–virus interactions. Additionally, the emerging concept of virome dysbiosis as a driver of chronic intestinal inflammation in IBDs presents promising avenues for therapeutic intervention. Research efforts focusing on virome transplantation and phage therapy hold significant potential in modulating the dysbiotic virome and restoring gut microbiome homeostasis in IBD patients [91,111,113,117]. However, further studies are warranted to elucidate the safety, efficacy, and long-term outcomes of these novel therapeutic approaches. In light of these findings, future research endeavors should prioritize elucidating the intricate mechanisms by which viral infections and virome dysbiosis contribute to immune-mediated gastrointestinal diseases. By advancing our understanding of these processes, we can pave the way for the development of targeted interventions aimed at preventing disease onset and alleviating symptomatology in affected individuals, ultimately improving patient outcomes and quality of life.

## Figures and Tables

**Table 1:** Virus in celiac disease: mechanisms implicated in disease pathogenesis.

Virus	Pathogenetic Mechanism	References	Subjects
Rotavirus	Molecular mimicry Augmenting mucosal permeability	[17] [39] [43]	H H H
Reovirus	Th1 response against dietary antigen	[14] [44] [46]	A A A
Norovirus	Th1 response against dietary antigen	[48]	A
Enterovirus	Inflammation of small intestine heightens tissue TGA levels	[49] [53]	H H
EBV	Activation of inflammatory cells promotes refractory CD	[54]	H
CMV	Increasing Vδ1+ cells, protective role?	[55]	H

EBV: Epstein–Barr virus; CMV: Citomegalovirus; H: humans; A: animals.

**Table 2:** Viruses in achalasia: mechanisms implicated in disease pathogenesis.

Virus	Pathogenetic Mechanism	References	Subjects
HSV-1	Th1 response against myenteric neurons	[18] [64] [68]	H H H
VZV	Chronic infection in esophageal neurons leads to alteration in esophageal motility	[19] [21]	H H
HIV	Loss of autonomic innervation Esophageal opportunistic infection in AIDS	[69] [70] [71]	H H H

HSV-1: Herpes Simplex Virus 1; VZV: Varicella Zoster Virus; HIV: Human Immunodeficiency Virus; H: humans.

## Data Availability

No new data were created or analyzed in this study. Data sharing is not applicable to this article.
